# The Hepcidin/Ferroportin axis modulates proliferation of pulmonary artery smooth muscle cells

**DOI:** 10.1038/s41598-018-31095-0

**Published:** 2018-08-28

**Authors:** Latha Ramakrishnan, Sofia L. Pedersen, Quezia K. Toe, Laura E. West, Sharon Mumby, Helen Casbolt, Theo Issitt, Benjamin Garfield, Allan Lawrie, S. John Wort, Gregory J. Quinlan

**Affiliations:** 1 Vascular Biology Group, National Heart and Lung Institute, Imperial College London, Faculty of Medicine, Guy Scadding Building, London, SW3 6LY UK; 2 Department of Infection, Immunity & Cardiovascular Disease, University of Sheffield, S10 2RX Sheffield, UK

## Abstract

Studies were undertaken to examine any role for the hepcidin/ferroportin axis in proliferative responses of human pulmonary artery smooth muscle cells (hPASMCs). Entirely novel findings have demonstrated the presence of ferroportin in hPASMCs. Hepcidin treatment caused increased proliferation of these cells most likely by binding ferroportin resulting in internalisation and cellular iron retention. Cellular iron content increased with hepcidin treatment. Stabilisation of ferroportin expression and activity via intervention with the therapeutic monoclonal antibody LY2928057 reversed proliferation and cellular iron accumulation. Additionally, IL-6 treatment was found to enhance proliferation and iron accumulation in hPASMCs; intervention with LY2928057 prevented this response. IL-6 was also found to increase hepcidin transcription and release from hPASMCs suggesting a potential autocrine response. Hepcidin or IL-6 mediated iron accumulation contributes to proliferation in hPASMCs; ferroportin mediated cellular iron excretion limits proliferation. Haemoglobin also caused proliferation of hPASMCs; in other novel findings, CD163, the haemoglobin/haptoglobin receptor, was found on these cells and offers a means for cellular uptake of iron via haemoglobin. Il-6 was also found to modulate CD163 on these cells. These data contribute to a better understanding of how disrupted iron homeostasis may induce vascular remodelling, such as in pulmonary arterial hypertension.

## Introduction

Hepcidin is a small (25 amino acid) peptide hormone largely responsible for regulation of body iron homeostasis^1^. First identified in urine, hepcidin is predominantly produced by hepatocytes^2^ and when released into the circulation is able to interact with the membrane active cellular iron exporter ferroportin, causing it to be endocytosed, thereby preventing iron exit and encouraging cellular iron retention^3^. Together hepcidin and ferroportin currently represent the only known regulators of cellular iron export. Ferroportin is chiefly expressed in cells linked to iron uptake (from the diet) and homeostasis; examples include duodenal enterocytes, macrophages and hepatocytes.

Hepcidin expression is regulated by plasma iron levels and stores; this transcriptional control is facilitated by the bone morphogenetic protein receptor (BMPR) coupled SMAD signalling pathway^4^. Importantly, infection and inflammation also regulate the synthesis of hepcidin, a response most notably linked to IL-6 activation of the JAK/STAT pathway^5^. Resulting hypoferremia, described as the anaemia of inflammation, helps limit microbial virulence (reviewed in^6^). Consequences related to increased iron storage are likely to include deficient erythropoiesis and perturbation of cellular function related to excess iron accumulation^7,8^. In this regard, iron is also an essential requirement for cell proliferation; when available in excess, a proliferative state is encouraged^1,7,9^. Current perceptions suggest that most cell types express little or no ferroportin as iron is utilised for metabolic requirements alone and therefore there is no need to export this resource. However, new studies are emerging which indicate expression and or regulation of ferroportin and hepcidin linked to iron retention in cells of various cancer categories^10–12^ with iron retention being linked to cell survival and proliferation, thus suggesting the importance of this axis in proliferative disease.

Pulmonary artery hypertension (PAH) is a disease process in which abnormal iron homeostasis has also been implicated^13^ and hepcidin excess demonstrated^8^. It is characterised by regionalised hyperplasia of smooth muscle and endothelial cell components of resistance, pre-capillary pulmonary arterioles. Known as a rare disease, PAH is classified into idiopathic, heritable or forms resulting in association with specific conditions, such as connective tissue or congenital heart disease^14^. Genetic mutations, and in particular those related to BMPR II underscore most heritable cases and a significant proportion of sporadic cases of idiopathic PAH^15^. Inflammation may be the common link between dysfunctional BMP signalling and loss of iron; importantly plasma IL-6 is raised in patients with PAH^16^. Intriguingly, increased autophagy mediated by lysosomal action (where BMPR-II and ferroportin are both degraded) has been implicated in PAH^17^, indicating a link with altered iron handling.

As for the source of iron for cellular uptake, this would be most likely be provided via transferrin receptor-1 mediated mechanisms. However, the increasing recognition that free haemoglobin is associated with PAH^18^, without the classical haemolytic disease phenotype, may suggest additional avenues for iron acquisition by pulmonary vascular cells.

The present study was therefore undertaken to evaluate whether there is any role for the hepcidin/ferroportin axis in proliferative responses of pulmonary artery smooth muscle cells. The aims of the study were threefold. Firstly, to describe for the first time the presence of the iron export protein, ferroportin in these cells. Secondly, to modulate ferroportin expression/activity in these cells to evaluate any subsequent influence on proliferative responses. Thirdly, to assess any role for free haemoglobin in proliferation and potential mechanisms for cellular uptake. These studies may provide insight into the potential role of disrupted iron homeostasis in vascular remodelling, such as observed in PAH.

## Results

### Ferroportin is expressed in hPASMCs and regulated by hepcidin

**Figure 1:**
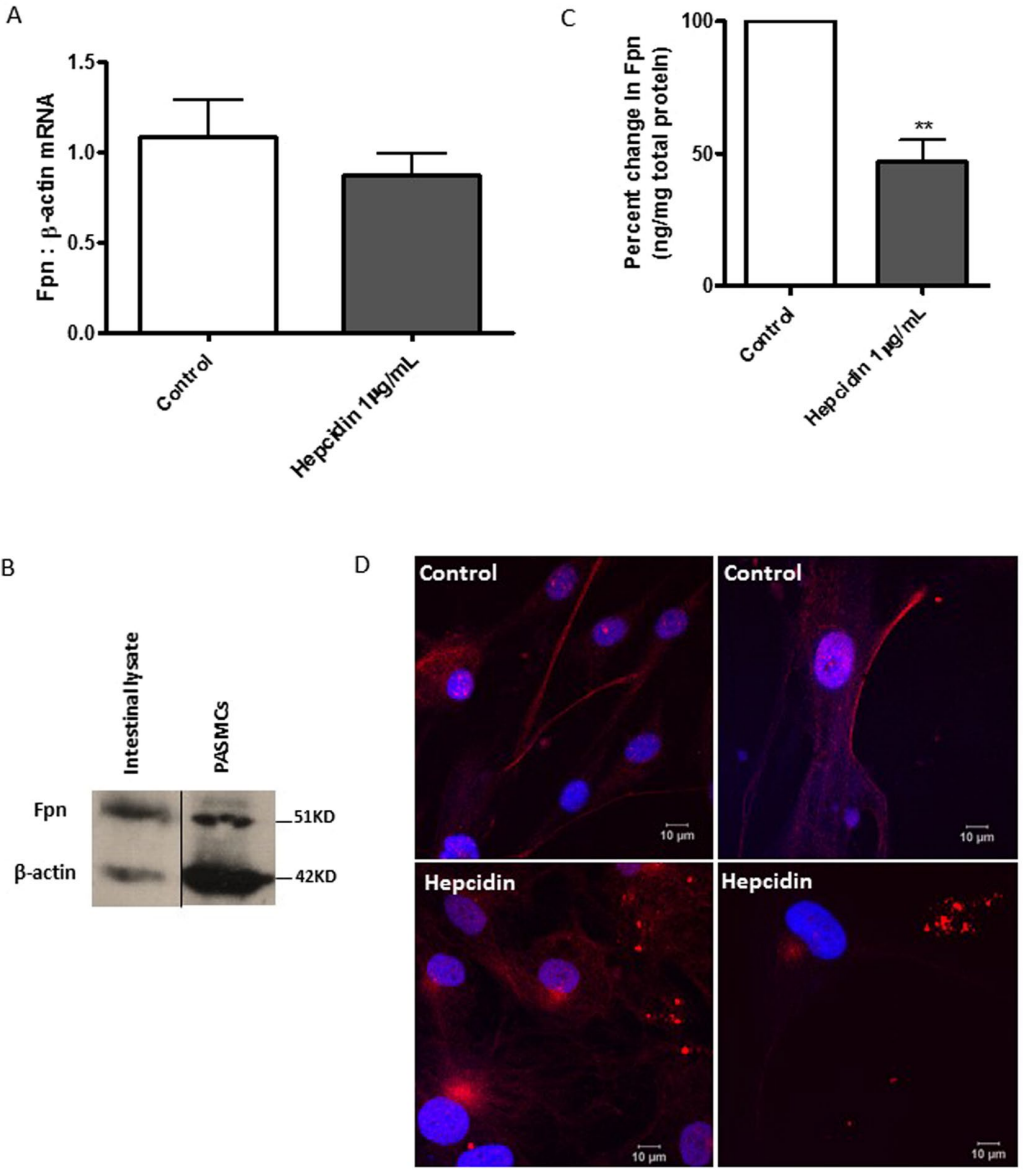
Ferroportin is expressed in hPASMCs and regulated by hepcidin. Confluent hPASMCs were (**A**) either mock-treated or treated with 1 µg/mL hepcidin for 2.5 h and total RNA extracted using RNeasy kit, cDNA synthesised using oligo-dT primers and RT-PCR performed using SYBR green with human Fpn (ferroportin) primers and β-actin as housekeeping gene. The values were further normalised as fold changes to the control untreated cells at time zero. N = 4 (**B**) mock-treated for 24 h, cells lysed and total protein extracted, quantified by Bradford assay and 40 µg of protein separated on 10% SDS-PAGE and transferred onto nitrocellulose membranes. Western blotting was performed using Rabbit anti-Fpn as primary and Goat anti-rabbit IgG conjugated with horse-radish peroxidase as secondary antibodies. Human intestinal lysates (Abcam) were used as positive control. N = 3 (**C**) either mock-treated or treated with 1 µg/mL hepcidin for 24 h, cells lysed and total protein extracted. Fpn expression was quantitated using an ELISA kit (BlueGene Biotech) and normalised to total protein estimated by Bradford reagent. N = 4 (**D**) Confocal images of hPASMCs grown with (top panels) normal media or (bottom panels) treated with 1 µg/mL hepcidin for 20–22 h and immuno-stained with rabbit anti-Fpn antibody and goat anti-rabbit IgG secondary antibody tagged with Alexa-568. The cells were further counterstained with DAPI and images captured using Leica LSM 510 confocal microscope. Scale bar = 10 µm; N = 5. Student’s t test was performed; **p < 0.01.

Ferroportin mRNA and protein expression is demonstrated in human pulmonary artery smooth muscle cells (hPASMCs) (Fig. 1). Basal ferroportin mRNA expression was detectable in control hPASMCs. Treatment of hPASMCs with hepcidin (1 µg/ml) for 2.5 hours had no significant effect on the levels of mRNA expression (Fig. 1A). Western blot analysis of cell lysates from control hPASMCs revealed a band of approximately 50 kD corresponding to ferroportin when compared against the standard human intestinal lysates (Fig. 1B). A high sensitivity ELISA was employed for the quantification of ferroportin levels. Basal ferroportin protein levels were markedly attenuated 24 h after hepcidin treatment, suggesting a post-translational regulatory control mechanism (Fig. 1C). Further, immunocytochemistry followed by confocal microscopy demonstrated cell surface localisation as well as intracellular distribution of ferroportin in unstimulated cells (Fig. 1D, upper panels). Hepcidin treatment caused a shift in distribution of ferroportin away from the cell surface, towards punctate intracellular localisation, suggestive of vesicular clustering (Fig. 1D, lower panels). The size of these punctate bodies varied from 0.5–1 µM, which is consistent with the size of lysosomes, suggesting a potential site for degradation of endocytosed ferroportin. In relation to these findings, in initial studies using lung samples from rat models of PAH (monocrotaline and sugen hypoxia), very low levels of diffuse ferroportin staining was seen in smooth muscle cells, with additionally a few cells staining strongly in the moncrotaline sample, most likely monocytes. Beyond indicating some presence of ferroportin in these relevant cells it is not possible to interpret these findings further. However, in this this regard there was a marked decrease in ferroportin staining in sugen hypoxia spleen (positive control) when compared to control spleen, which is suggestive of a global hepcidin response in this model of PAH, see Supplemental data Fig. 1.

### IL-6 increased hepcidin expression and down-regulated ferroportin in hPASMCs

**Figure 2:**
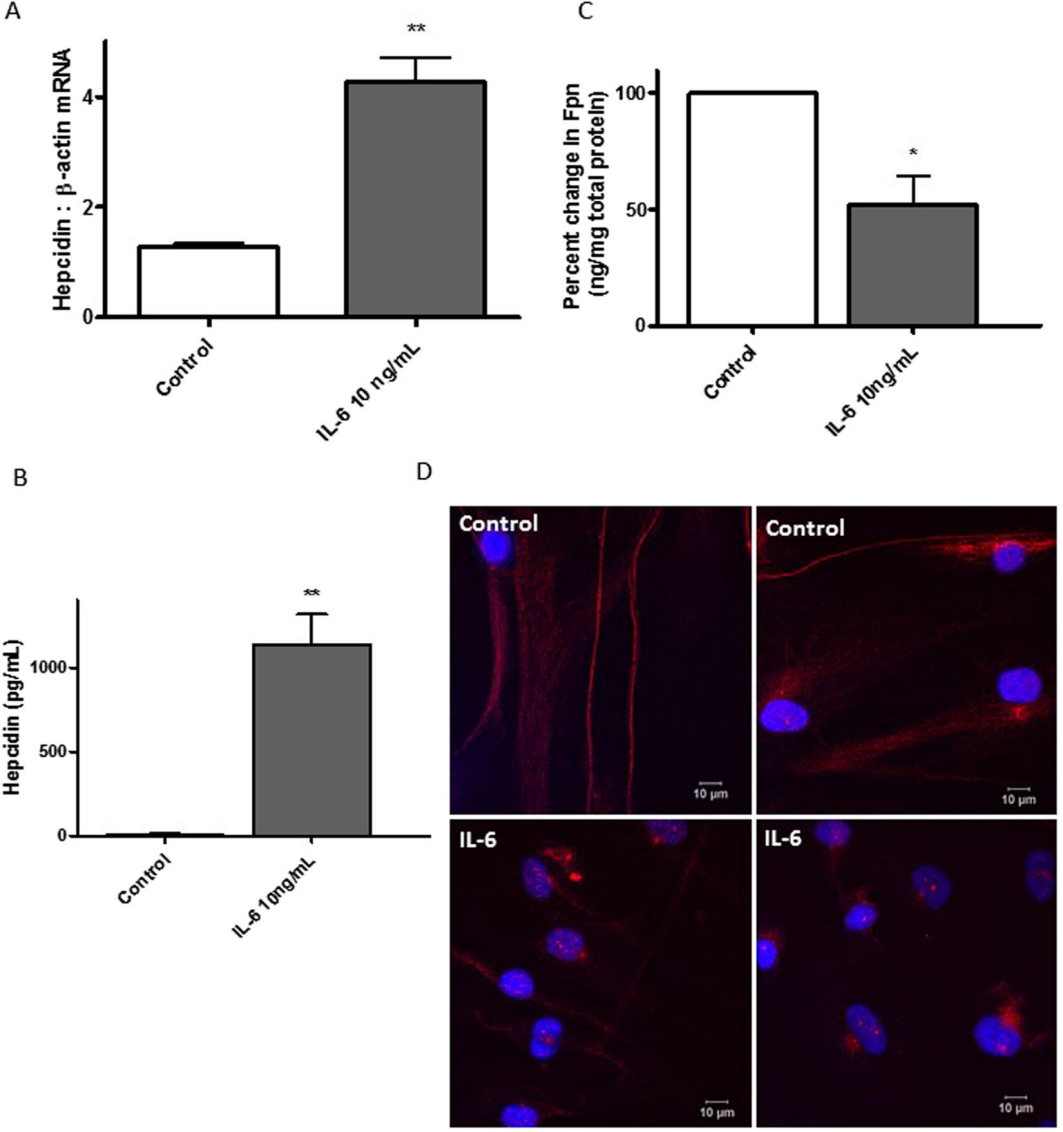
IL-6 increased hepcidin expression and down-regulated ferroportin in hPASMCs. Confluent hPASMCs were either mock-treated or treated with 10 ng/mL IL-6 (**A**) for 2.5 h and total RNA extracted using RNeasy kit, cDNA synthesised using oligo-dT primers and RT-PCR performed using SYBR green with human hepcidin (Hamp-1) primers and β-actin as housekeeping gene. The values were further normalised as fold changes to the control untreated cells at time zero. N = 4 (**B**) for 24 h, media supernatant collected and hepcidin secretion was quantitated using an ELISA kit (R&D Systems). N = 4 (**C**) for 24 h cells lysed and total protein extracted. Ferroportin expression was quantitated using an ELISA kit (BlueGene Biotech) and normalised to total protein estimated by Bradford reagent. N = 4 (**D**) Confocal images of hPASMCs grown with (top panels) normal media or (bottom panels) treated with 10 ng/mL IL-6 for 20–22 h and immuno-stained with rabbit anti-Fpn antibody and goat anti-rabbit IgG secondary antibody tagged with Alexa-568. The cells were further counterstained with DAPI and images captured using Leica LSM 510 confocal microscope. Scale bar = 10 µm, N = 5. Student’s t test was performed; **p < 0.01; *p < 0.05.

IL-6 is a known regulator of hepcidin expression^5^ and, therefore, iron homeostasis. IL-6 treatment of hPASMCs clearly demonstrated that both mRNA and protein secretion of hepcidin was significantly up regulated by 2 hours and 24 hours respectively (Fig. 2A,B). Determined as a fold change compared to the housekeeping gene β-actin, hepcidin expression increased 3.3 fold with 10 ng/ml IL-6. Conversely, 48 hours after IL-6 treatment, ferroportin protein was significantly decreased by 48% compared to control values (p < 0.01, Fig. 2C). In addition, IL-6 treatment was able to decrease the cell surface expression of membrane-bound ferroportin, as well as to alter the distribution of intra-cellular ferroportin similar to that seen with hepcidin treatment (Fig. 2D).

### Hepcidin and IL-6 stimulated proliferation of hPASMCs is inhibited by LY2928057

**Figure 3:**
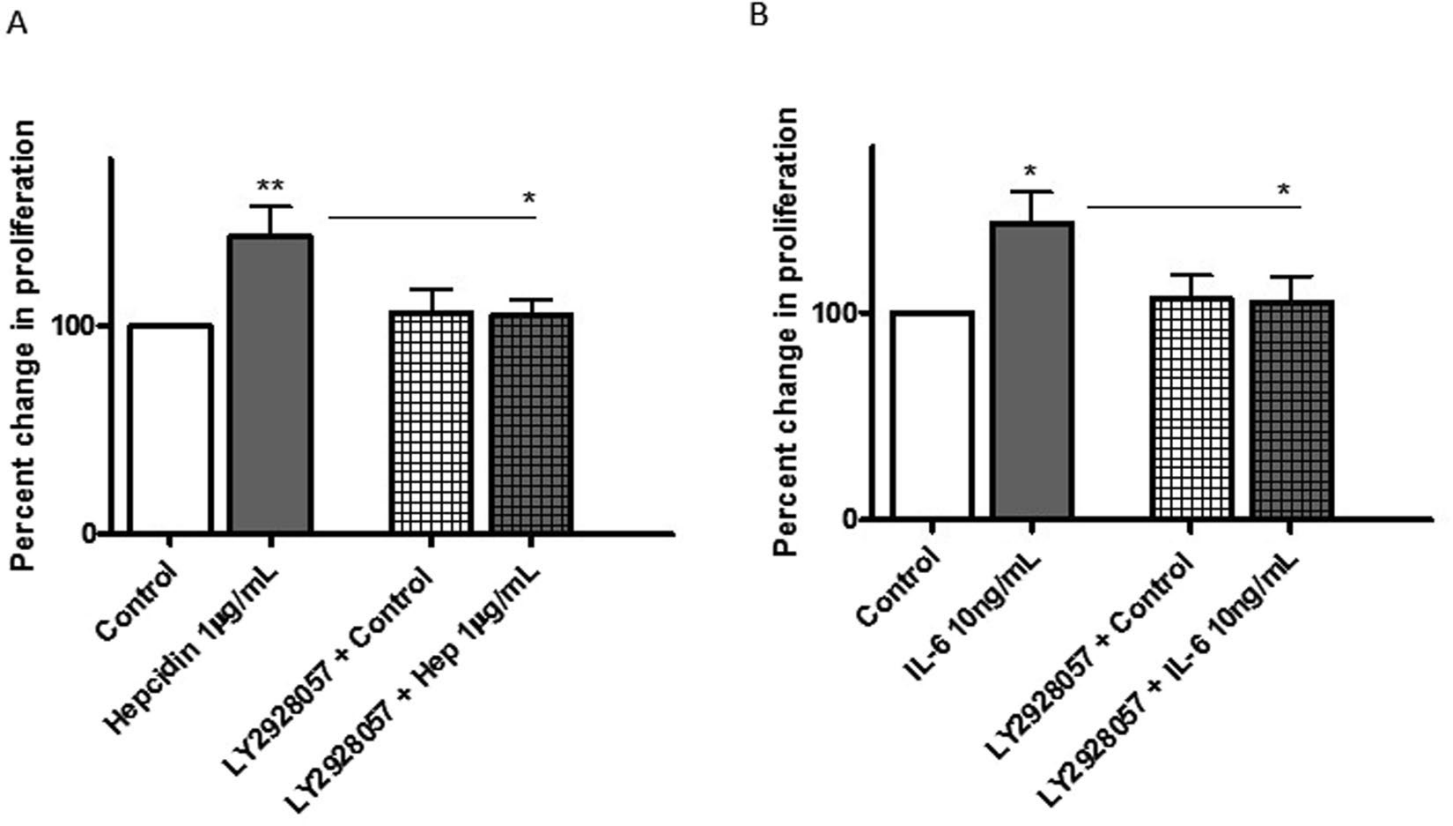
Hepcidin and IL-6 stimulated proliferation of hPASMCs is inhibited by LY2928057. hPASMCs were plated on to 96 well plates (2500 cells/well). After initial adherence, the cells were serum starved for 20–24 h prior to pre-treatment with either medium alone or (**A**) 1 µg/mL hepcidin or (**B**) 10 ng/mL IL-6 for 24 h. Following that the cells were either exposed to 1 μg/mL LY2928057 or media alone for 1.5 h followed by further repeat challenge with hepcidin or IL-6 at indicated concentrations (or media alone) for 24 h. BrdU was introduced (at manufacturer recommended concentration) for a further 24 h before harvesting the plates. Proliferation was quantified by BrdU ELISA kit using anti-BrdU-POD antibody. All treatments were done in triplicates. All readings were normalised to the control untreated cells. Data shown are ± SEM. N = 4. ANOVA followed by Bonferroni post hoc test was performed; *p < 0.05; **p < 0.01.

Hepcidin treatment (1 µg/ml) resulted in a significant increase in the proliferation of hPASMCs (Fig. 3A, left panels). Compared to controls, treatment with 1 µg/ml hepcidin caused a 43% increase in proliferation. However, pre-treatment of hPASMCs with the monoclonal ferroportin antibody LY2928057, (that stabilises membrane expression and iron export activity in spite of hepcidin availability)^19^, was able to significantly prevent the hepcidin driven proliferative response. LY2928057 pre-treatment restricted proliferation of hepcidin treated cells to 5% (Fig. 3A, right panels). Furthermore, IL-6 treatment also significantly enhanced hPASMC proliferation at 10 ng/mL treatment concentrations versus untreated cells (Fig. 3B, left panels). 10 ng/mL IL-6 increased proliferation of hPASMCs by 43% respectively. Importantly, pre-treatment with LY2928057 blocked the proliferative response to IL-6 (Fig. 3B, right panels).

### Hepcidin and IL-6 increased total iron content reversed by LY2928057

**Figure 4:**
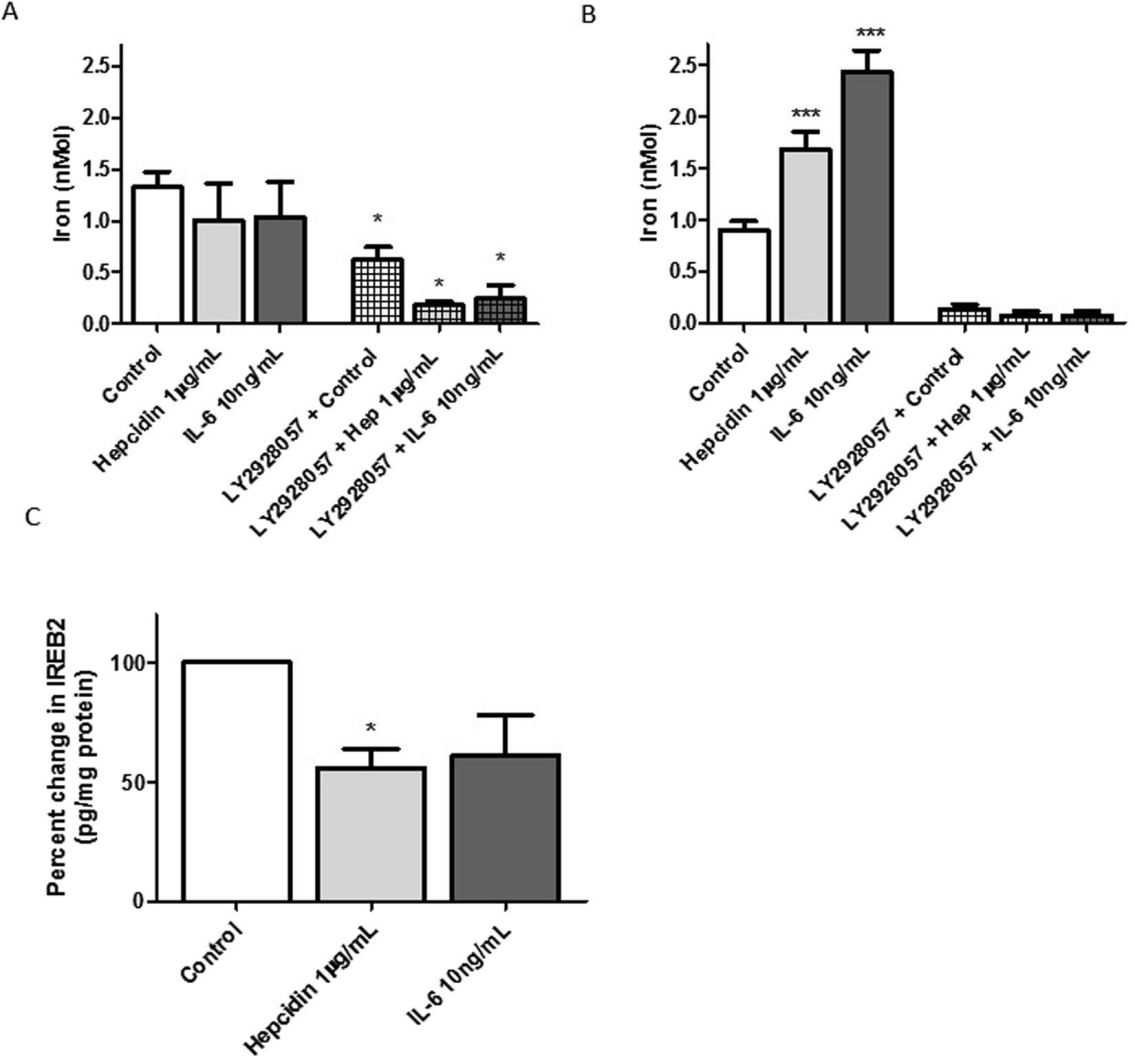
Hepcidin and IL-6 increased total iron content reversed by LY2928057. hPASMCs were plated on to 12 well plates (0.75 × 10^5^ cells per well). After adherence, the cells were pre-treated with either medium alone or 1 μg/mL LY2928057 for 1.5 h. Following that the cells were either exposed to 1 µg/mL hepcidin or 10 ng/mL IL-6 or media alone with continued presence (or absence in controls) of 1 μg/mL LY2928057 for a further (**A**) 24 or (**B**) 48 h. Total iron content in the cell lysate was quantitated using Colorimetric Iron Assay kit from Abcam. N = 3 (**C**) Confluent hPASMCs on 6 well plates were either treated with media alone or 1 µg/mL hepcidin or 10 ng/mL IL-6 in the presence of 0.2% FCS for 24 h cells. The cells were lysed using cell lysis buffer and total protein extracted. IREB2 expression was quantitated using an ELISA kit (Aviva) and normalised to total protein estimated by Bradford reagent. N = 4. ANOVA followed by Bonferroni post hoc test (**A**,**B**), or Dunn’s post hoc test (**C**) was performed; *p < 0.05; ***p < 0.001.

The hepcidin/ferroportin axis regulates cellular iron content and the role for iron in cellular proliferation^20^. Experiments were therefore undertaken in order to ascertain if hepcidin and/or IL-6 treatment favoured iron retention in hPASMCs and secondly if stabilisation of ferroportin expression with LY2928057 was able to limit such accumulation. Utilising a colourimetric iron assay, which measures total iron (Fe^3+^ and Fe^2+^) there was no measureable increase in iron in cells treated with either hepcidin or IL-6 for 24 hours (Fig. 4A, left panels). However pre-incubation with LY2928057 resulted in a significant decrease in cellular iron levels: 5.3 fold for hepcidin and 4.16 fold for IL-6 versus 2.14 fold for untreated cells (Fig. 4A, right panels). Interestingly, undertaking the same measurements at 48 hours revealed significant increases in cellular iron content: 1.88 fold and 2.72 fold for cells treated with hepcidin and IL-6 respectively (Fig. 4B, left panels) even though the measurable cellular iron diminished beyond the limits of assay measurement in LY2928057 pre-treated cells (Fig. 4B, right panels). In further support, levels of Iron Regulatory Protein 2 (IREB2), a surrogate for iron levels, declined in cells treated with either hepcidin or IL-6 and significantly so for hepcidin treated cells (Fig. 4C).

### CD163 is expressed in hPASMCs and regulated by Hb and IL-6

**Figure 5:**
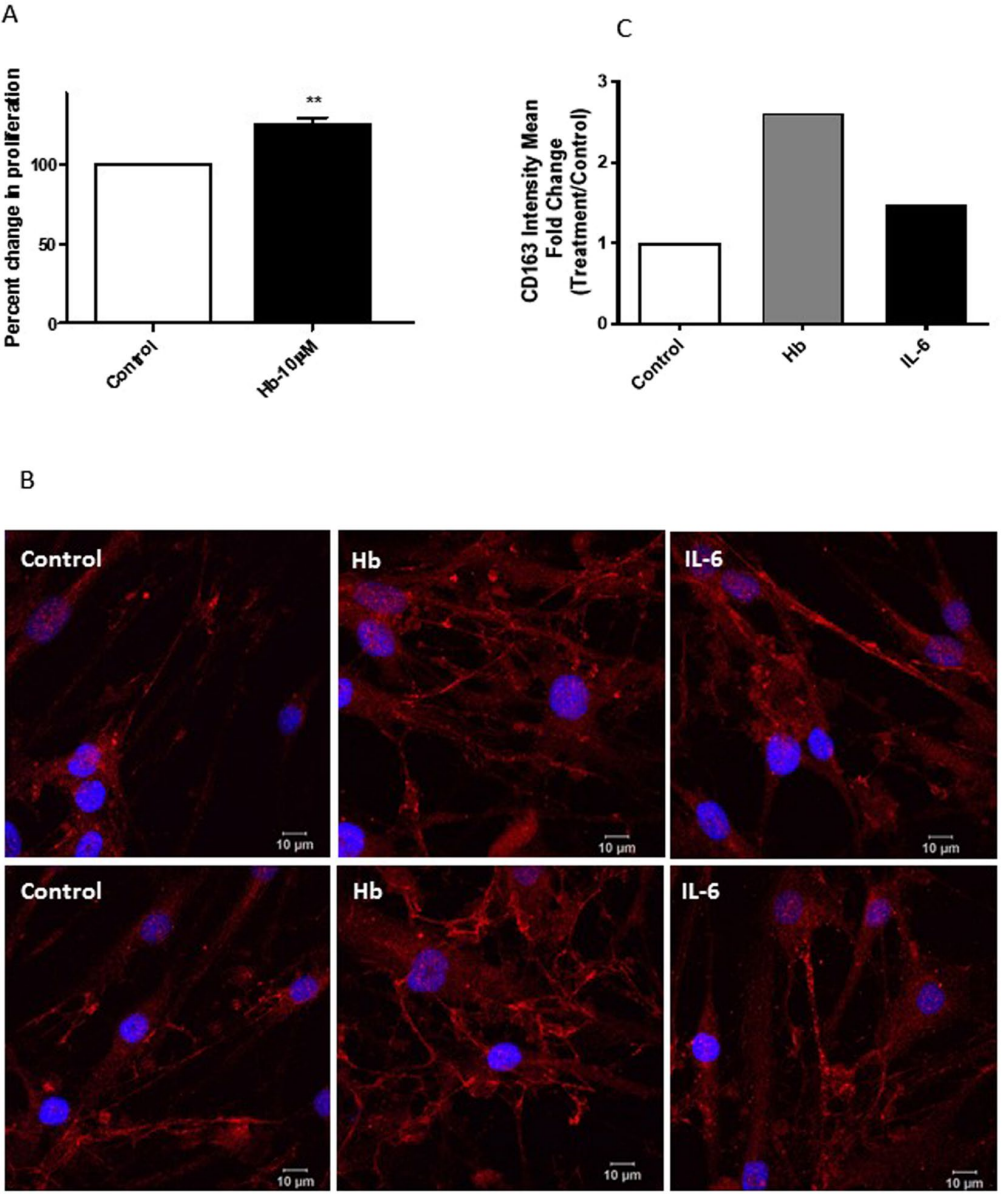
CD163 is expressed in hPASMCs and regulated by Hb and IL-6. (**A**) hPASMCs were plated on to 96 well plates (2500 cells/well). After initial adherence, the cells were serum starved for 20–24 h prior to pre-treatment with either medium alone or 10 µM haemoglobin (Hb) for 2.5 h followed by fresh medium replacement and allowed to grow for 24 h. BrdU was introduced (at manufacturer recommended concentration) for a further 24 h before harvesting the plates. Proliferation was quantified by BrdU ELISA kit using anti-BrdU-POD antibody. All treatments were done in triplicates. All readings were normalised to the control untreated cells. Data shown are ± SEM. N = 6. Student’s t test was performed; **p < 0.01. (**B**) Confocal images of hPASMCs grown with normal media (left panels) or treated with either 10 µM Hb (middle panels) for 2.5 h followed by normal media for 20 h or 10 ng/mL IL-6 (right panels) for 22 h and immuno-stained with rabbit anti-CD163 antibody and goat anti-rabbit IgG secondary antibody tagged with Alexa-568. The cells were further counterstained with DAPI and images captured using Leica LSM 510 confocal microscope. Scale bar = 10 µm. (**C**) Analysis of CD163 expression by immunofluorescence. Confocal images were analysed for integrated density, minus background and CD163 normalised against DAPI. This has then been expressed as fold change relative to control cells. Region of interest was defined by DAPI staining +25%.

A brief exposure of hPASMCs to 10 µM haemoglobin resulted in a significant increase (25%) in the proliferation of these cells (Fig. 5A). Basal mRNA expression of the haemoglobin/haptoglobin receptor molecule CD163 was detectable by RT-PCR (data not shown). Further, immunocytochemistry followed by confocal microscopy demonstrated cell membrane as well as intracellular expression of CD163 in unstimulated cells (Fig. 5B, left panels). Haemoglobin treatment markedly increased the expression of CD163 (Fig. 5B, middle panels) whilst IL-6 caused both an increase in the expression as well as a shift in the distribution of CD163 as punctate vesicles (Fig. 5B, right panels). Confocal images were further analysed for integrated density, minus background and CD163 normalised against DAPI. This has then been expressed as fold change relative to control cells. Increased CD163 expression is seen in both haemoglobin and IL-6 treated cells relative to control, statistics were deemed to be appropriate for these data. (Fig. 5C).

## Discussion

This study has established for the first time the presence of the cellular iron exporter ferroportin in hPAMSCs and an operational role for this protein in the control of iron content in these cells; being linked to interaction with the iron regulatory peptide hepcidin. When elevated levels of hepcidin are present, interaction with ferroportin ensues resulting in internalisation of the complex and degradation, as confocal imagery and ELISA based studies indicate. Moreover this set of circumstances enhances iron retention, a finding that is further supported by the observed decline in cellular iron regulatory protein 2 (IREB2) levels; IREB2 levels are regulated by cellular iron content and decrease in the presence of iron^21^. In addition, under these circumstances hPASMC proliferate more, which suggests that iron retention encourages a proliferative state. In support of this notion, pre-treatment of hPASMCs with the therapeutic monoclonal antibody LY2928057 inhibited this hepcidin driven proliferative response and importantly also prevented cellular iron accumulation.

Additional studies have demonstrated similar responses in hPASMCs to those reported above, in response to IL-6 treatments. Importantly IL-6 was also shown to increase hepcidin mRNA levels and hepcidin release from hPASMCs eliciting the possibility of an autocrine hepcidin response in these cells; although further studies will be required to confirm any functionality related to this observation. However, such a role for hepcidin has been suggested previously in cardiomyocytes^22^. These novel observations are intriguing and potentially indicate transcriptional control of, and a novel source of, hepcidin synthesis in hPASMCs which may be further linked to an autocrine aspect of cellular iron regulation/cellular proliferation in this cell type. It is likely that these IL-6 mediated responses are signalled via the canonical Jak/stat3 pathway; however, given the novelty of these observations a fresh examination of this and other potential signalling pathways seems warranted.

The hepcidin/ferroportin axis is a well-established regulator of cellular iron content and the role of IL-6 in cellular iron regulation is also well established; however, the operation of these regulatory mechanisms has not previously been reported in hPASMCs. Moreover, the relationship between cellular iron accumulation, these axes and proliferation has also not been described previously for this cell type. Interestingly, disordered hepcidin/ferroportin activity have been shown to promote growth of breast cancer cells, with elevated ferroportin expression responsible for inhibition of proliferation; importantly IL-6 was also shown to promote proliferation of these cells^23^, supporting the above concept. These findings are also intriguing and potentially of relevance to pulmonary arterial hypertension not least because of the excessive and unrestricted PASMC proliferation and pulmonary vascular remodelling characteristic of this condition. Additionally, there is emerging evidence indicating disrupted iron homeostasis^13^, elevated blood levels of hepcidin^8^, IL-6^16^ and associations with genetic abnormality (BMPRII) in heritable and sporadic PAH and regulation of hepcidin expression may be of mechanistic relevance in PAH.

A role for decompartmentalised haemoglobin in PAH is established for haemolytic diseases processes^24^ and is thought by some to be driven by nitric oxide scavenging and consequent vaso-restriction^25^. In other forms of PAH any association with free haemoglobin is less well established, but emerging studies in patients with iPAH and heritable PAH have demonstrated enhanced levels of zinc - protoporphyrin^26^ and elevated creatine^27^ as indicators of subclinical haemolysis in these patient groups. Moreover, in another study, plasma free haemoglobin, was independently associated with increased risk for admission to hospital in PAH groups^28^. Furthermore, decreased levels of haptoglobin are seen in PAH phenotypes suggesting increased haemoglobin binding and removal in these patients^29^ which is likely related to enhanced or on-going haemolysis. Studies presented herein suggest that free haemoglobin is also a pro-proliferative agent for hPASMCs and that the first description of the presence of CD163 on these cells suggests a means for cellular uptake of this iron containing protein complex. Whilst not directly associated with the activity of the hepcidin/ferroportin axis it is noteworthy that in addition to haemoglobin itself, IL-6 a known regulator of hepcidin, also seems to modulate the expression of CD163 in these cells, which may suggest some complementary activity linked to iron uptake and retention. To give some context to these preliminary studies, in a recently published study using an *in vivo* model of vascular remodelling driven by cellular iron accumulation the process was reversed with haptoglobin therapy^30^ suggesting a potential role proliferative role for free haemoglobin in this model and supporting our findings.

Taken as a whole our observations are further supported by several recent studies that have linked vascular remodelling with iron availability: firstly, administration of the iron chelator, desferrioxamine to rats inhibited chronic hypoxia induced pulmonary hypertension and remodelling; secondly, the same authors showed that the iron chelators, hinokitiol and HBED inhibited proliferation of cultured pulmonary artery smooth muscle cells^31^; thirdly, iron was found to induce remodelling in cultured rat pulmonary artery endothelial cells^32^; and fourthly, plumbagin, a known iron chelator^33^ was found to limit proliferation in hPASMCs and to decrease distal PA remodelling in a rat model of PAH^34^. Conversely, monocrotaline treated rats fed an iron restricted diet were less prone to pulmonary vascular remodelling and right ventricular failure; interestingly, hepcidin levels decreased in these animals compared to those on a normal iron containing diet, suggesting that cellular iron retention is less likely in these circumstances^35^. There is evidence from clinical studies that suggest iron supplementation can prove of benefit to certain PAH patient groups^36,37^; all of which may seem to be at variance with our findings. However, considerations based on short-term improvement in patient wellbeing may need to be balanced against potential longer terms effects related to iron accumulation. Nevertheless, what is emerging from these various study counterpoints is that iron homeostatic control is important at the level of the pulmonary vasculature and that insufficient or excessive iron availability may contribute to vascular dysfunction; for a recent review see^38^.

We have not investigated in detail potential mechanisms related to iron accumulation that may lead to proliferative responses in hPASMCs. However, it is plausible to suggest candidates given current understanding of cellular iron metabolism. Mitochondria are centres for iron uptake and utilisation, and are subject to functional perturbation linked to iron deficits or overload^39^; importantly mitochondrial respiratory rate and hydrogen peroxide production and release into the cytosol are transcriptional signals for an array of processes including for cell cycle progression and proliferation, reviewed in^40^. In general terms a depressed mitochondrial respiratory rate favours proliferation. Iron loading of mitochondria will affect respiration directly thereby potentially promoting a pro-proliferative state. Moreover, increased levels cytosolic ferrous iron (Fe^2+^) will catalytically remove hydrogen peroxide thereby influencing redox signalling pathways which again may promote proliferation. In addition, iron loading of mitochondria may well favour switching to mitochondrial glycolytic pathway predominance, in common with cancer phenotypes and which has also been implicated in PAH, reviewed in^41^. The transcription factor Hypoxia inducible factor (hif) and its role in cell proliferation have featured in much recent PAH related research. Iron and oxygen are known repressors of Hif assembly, whereas hypoxia or iron deficits allow for assembly, migration to the nucleus and gene transcription^42^. Given that the current study was undertaken in normoxia and in cells with increased iron loading one could speculate that Hif was unlikely to be involved, however this may be somewhat of an oversimplification as iron has numerous effects in cells beyond inhibition of hif activity. Indeed, there are numerous reports of normoxic hif activity and iron related effects on cellular redox status including of mitochondria may well favour hif activation^41^, so a role for hif cannot be excluded. The iron regulatory proteins direct cellular iron homeostatic control^43^ with IRP-2 assuming the dominant role in these processes. Recent studies have implicated sustained IRP-2 activity in cancer cell proliferation^44,45^. For the current study measurement of IRP-2 was used as a surrogate for cellular iron. Levels did decline in response to hepcidin intervention and associated iron retention, but it is noteworthy that this experimental model was unable to abolish IRP-2 completely which may be of functional relevance given the emerging role for this protein complex in proliferation. Clearly cellular iron fluxes can affect many intracellular responses and interactions including those described above, establishing greater mechanistic insight into these processes seems appropriate.

Our studies may offer some explanation as to the aetiology of PASMC proliferation that may be of relevance to PAH onset and progression and, potentially a therapeutic option via intervention to target the hepcidin/ferroportin axis, or via IL-6 antagonists. However, further studies will be required to investigate these processes in greater depth. These will include the use of ferroportin over-expression or knock-down protocols to mimic the actions of LY2928057 or hepcidin and thereby strengthen the findings reported herein. Moreover there are several other limitations to our study: firstly, as mentioned above detailed mechanistic studies are required; secondly, we have conducted the experiments with hPASMCs from normal donors (not PAH patients); thirdly, we have not as yet investigated these proliferative processes *in vivo;* and fourthly, we have not examined the effects of hypoxia on these responses, hypoxia being of relevance to PAH. Finally, given the novelty of our findings no evaluation of ferroportin expression or otherwise has to our knowledge as yet been undertaken in subjects with or samples from patients with PAH nor indeed has any evaluation of CD163.

In spite of these shortfalls, we would suggest that these studies offer avenues for further research into the mechanisms of PASMC proliferation in PAH, and potentially in other wider aspects of vascular dysfunction, where disrupted iron homeostasis linked to the hepcidin/ferroportin axis that is responsible for either iron accumulation or iron deficits may be of importance.

## Methods

### Cells and reagents

Human pulmonary smooth muscle cells (hPASMCs) were either isolated from samples of pulmonary artery (the order of vessels within the specimens is approximately 4th to 6th branching although some may be smaller, depending on where the resection sample is taken) from patients undergoing lung lobectomy at the Royal Brompton Hospital and maintained in Dulbecco’s Modified Eagle’s Medium (DMEM) supplemented with 15% fetal calf serum, 2 mM glutamine, 100 U/ml penicillin, and 100 μg/ml streptomycin. In order to select only hPASMCs for study from cells isolated from pulmonary arteries. hPASMCs were selected in high serum DMEM over a very long period of time thereby excluding other cell types and characterised by morphological examination; for examples see Supplementary data Fig. 2. Only those cells that had a typical long and narrow spindle shape (with mono nucleus in the centre of the cell) and which formed a uniform, continuous monolayer were propagated further. Only cells that maintained this morphology (over passage 4–8) were used. Any arteries that produced cells with other random morphologies (fatter, non-spindle shape etc) were discarded. Those cells supplied from Promocell were provided fully characterised. Staining for smooth muscle cell markers SMA, SM-22 and SM-MHC was also undertaken, see Supplemental data Fig. 3A–C for examples of donor cells, and Supplemental data Fig. 4 for cells obtained from promocell. All characterised cells were maintained in smooth muscle cell (SMC) growth medium 2 (Promocell). Cells from a total of seven subjects were used for these studies, with at least three subjects used for each experimental protocol. Levels of ferroportin expression in hPASMCs as viewed by immunohistochemistry were relatively uniform across the donor pool whereas CD163 expression was subject to greater donor variability. General reagents were from Sigma (Dorset, UK) or Invitrogen (Paisley, UK). Hepcidin - 25 was purchased from Peptides International and IL-6 from R&D Systems. LY2928057 was kindly gifted by Eli-Lily. Rabbit anti-ferroportin antibody was purchased from Novus Biologicals and rabbit anti-CD163 from Santa Cruz Biotechnology. Haemoglobin (Met) was from Sigma-Aldrich.

### Primary cell culture and treatments

A 90% confluent hPASMC monolayer was trypsinised using detach kit (Promocell) as per manufacturer’s instructions. Briefly, the monolayer was swiftly washed with HEPES, treated with 0.025% Trypsin/0.01 EDTA solution to detach the cells followed by quenching with TNS (Trypsin Neutralising Solution). For culturing and passaging, the cells were plated at 7500 per sq.cm. Only cells from passage 4–7 were used in the experiments. For experiments, the cells were seeded on 6 well plates at 1.5 × 10^5^ cells per well. Cells were incubated with either media alone or 1 µg/mL hepcidin or 10 ng/mL IL-6 for 2.5 h for RNA extraction or 24 h or 48 h for protein extractions. For some experiments (confocal microscopy, IREB2 expression and hepcidin secretion), serum restricted media (DMEM supplemented with 0.2% fetal calf serum) was employed.

### RT-PCR

Total RNA was extracted using the RNeasy Mini kit (Qiagen) following the manufacturer’s instructions. RNA purity and concentration were measured by Nano-drop spectrophotometer. 0.6–1 μg of total RNA was used for reverse transcription to produce cDNA using oligo-dT primers (Invitrogen), 10 mM dNTP mix, RNasin plus (Promega) and M-MLV Reverse transcriptase enzyme (Invitrogen) according to manufacturer recommendations. Reverse transcriptase enzyme was omitted in negative controls. Real-time PCR using SYBR green was carried-out on a Rotor-Gene 6000 PCR machine using ferroportin and hepcidin (Qiagen) primers while β-actin was used as standard housekeeping gene. Both −RT and no template negative controls were employed. The change in expression was normalised to control untreated samples.

### Preparation of cell lysates

Cell lysis buffer (New England Biolabs) containing 20 mM Tris-HCl (pH 7.5), 150 mM NaCl, 1 mM Na_2_EDTA, 1 mM EGTA, 1% Triton, 2.5 mM sodium pyrophosphate, 1 mM β-glycerophosphate, 1 mM Na_3_VO_4_, 1 μg/ml leupeptin, and 1 mM PMSF was used to lyse the cells. Cells were scraped from the culture plates and centrifuged to remove debris. Total protein in cell lysates was determined by Bradford protein assay (Bio-Rad).

### ELISA

Ferroportin (BlueGene Biotech) and IREB2 (Aviva) from cell lysates and hepcidin (R&D Systems) from cell culture supernatants were quantified by ELISA according to the respective detailed manufacturer’s instructions. Ferroportin and IREB2 levels were normalised to total protein in the cell lysates.

### Western blot

Proteins were resolved on 4–12% gradient SDS-PAGE Tris gels (BioRad) and transferred to nitrocellulose membranes as described previously^46^. After blocking with 5% milk, membranes were probed with rabbit anti-ferroportin antibodies (1:1000). Human β-actin (New England Biolabs) was used as a loading control (1:2000). Further horseradish peroxidase-conjugated anti-rabbit IgG (New England Biolabs) was used as a secondary antibody (1:2000).

### Immunocytochemistry

hPASMCs were seeded on 8 well chamber slides at 10,000 cells per well with 15% DMEM. After initial adherence (20–22 h), the cells were treated with 0.2% DMEM alone or 1 µg/mL hepcidin or 10 ng/mL IL-6 (both made in 0.2% DMEM) for 48 hours followed by immuno-staining. Briefly, the cells were fixed using 100% freezing cold methanol for 10 minutes, followed by permeabilisation (0.2% Triton X-100 in PBS) for 5 minutes. The slides were then blocked with 1% BSA in PBS for an hour followed by incubation with rabbit anti-ferroportin or anti CD163 antibody (1:250) for another hour. A negative control without any primary antibody was also employed. Following four washes with 0.1% Tween-20 in PBS, the slides were incubated with 1:500 Alexa Flour 568 Goat anti-rabbit IgG for a further hour in dark. The cells were further counterstained with DAPI and images captured using Leica LSM 510 confocal microscope at a centralised facility at Imperial College London. For quantification of CD163 confocal images ImageJ was used. Cellular region of interest was determined by DAPI staining +25%. Integrated density was collected for CD163, minus background and normalised to DAPI. This was then expressed as fold change relative to control.

### Cell proliferation

The colorimetric BrdU cell proliferation assay kit (Roche) was used to measure cell proliferation. hPASMCs were plated on to 96 well plates (2500 cells/well). After adherence, the cells were serum starved (0.2% Serum) for 20–24 h prior to pre-treatment with either medium alone or 1 µg/mL hepcidin or 10 ng/mL IL-6 for 24 h. For treatments with 10 µM haemoglobin, the cells were replaced with fresh media after 2.5 h of Hb treatment. Following on, the cells were pre-incubated with LY2928057 (or mock-incubated) for 1.5 h prior to repeat treatments with either hepcidin or IL-6 for further 24 h. At this point, BrdU was introduced (at the manufacturer recommended concentration) for an additional 24 h before harvesting the plates. Proliferation was quantified using anti-BrdU-POD antibody, following the manufacturer’s instructions. All treatments were undertaken in triplicate.

### Iron assay

The total iron content of the cells was estimated by employing the Colorimetric Iron Assay kit (Abcam) as per the manufacturer’s guidelines. Firstly all Fe^3+^ was converted to Fe^2+^ which then reacted with Ferene S to produce a stable coloured complex with absorbance at 593 nm. Cu^2+^ interference was specifically blocked with the chelate chemical included in the buffer. 50 µl lysates were prepared in the iron assay buffer provided from treated hPASMCs grown on 6 well plates.

### Statistical analysis

Data is presented as ± SEM (standard error of mean) of the specified number of independent experiments. Statistical analysis was performed on GraphPad Prism. Student’s t test or ANOVA followed by Bonferroni post hoc test was employed to determine statistical significance. Level of significance achieved is indicated in individual figure legends.

### Ethics declaration

The use of normal lung tissue has been approved by the Royal Brompton and Harefield NHS Trust Research Ethics Committee (ethics number GQJW1). All procedures were carried out in accordance with the relevant guidelines and regulations. All patients gave written, informed consent prior to the use of their lung tissue.

## Electronic supplementary material

Supplementary information

## Data Availability

All data generated or analysed during this study are included in this published article.
