# Supplementary material for: The Hepcidin/Ferroportin axis modulates proliferation of pulmonary artery smooth muscle cells
Source: Sci Rep. 2018 Aug 28;8:12972. doi: 10.1038/s41598-018-31095-0 (PMC6113242; doi:10.1038/s41598-018-31095-0)
Supplement: Supplementary file 1 — Supplementary information [file 41598_2018_31095_MOESM1_ESM.pdf]

The Hepcidin / Ferroportin axis modulates proliferation of pulmonary artery smooth muscle cells

Latha Ramakrishnan[1], Sofia L Pedersen[1], Quezia K Toe[1], Laura E West [2], Sharon Mumby[1], Helen Casbolt [2] , Theo Issitt[1], Benjamin Garfield[1], , Allan Lawrie[2], S John Wort[1,3] , Gregory J Quinlan[1,3] *

1) Vascular Biology Group, National Heart and Lung Institute, Imperial College London, Faculty of Medicine, Guy Scadding Building, London SW3 6LY, UK.

2) Department of Infection, Immunity & Cardiovascular Disease, University of Sheffield S10 2RX, UK

3) These authors contributed equally to the production of this manuscript.

*Corresponding Author:

Email: g.quinlan@imperial.ac.uk

Supplementary Methods:

Animal treatment

Sprague Dawley rats were given single s/c injections of monocrotaline (60 mg/Kg; Sigma) or saline control followed by 3 weeks at normoxia. Wistar rats were given single s/c injections of SU5416 (20 mg/Kg; Tocris) followed by 3 weeks in hypobaric chambers equivalent to 18,000ft then 6 weeks at normobaric pressure. Control animals were not injected with SU5416 and kept at normobaric pressure throughout.

Immunohistochemistry

Ferroportin staining: Formalin-fixed paraffin-embedded (FFPE) tissue antigen was retrieved by heat-mediation in citrate buffer. The FFPE tissue was incubated with rabbit ant-SLC40A1 primary antibody (1:50; ab78066, Abcam) for 1 hour at room temperature. After, the tissue was incubated with biotinylated anti-rabbit secondary antibody (1:200; Vector Labs) for 30 minutes. Vectastain® ABC-HRP (Vector Laboratories) was applied and visualised by applying 3,3'-diaminobenzidine tetrahydrochloride (DAB). Tissue was counterstained with Carazzi's haematoxylin. Ferroportin is stained brown and nuclei are stained blue.

Iron staining: Ferric iron staining of (FFPE) was accomplished using the Iron Stain Kit (ab150674, Abcam). Briefly, the tissue was incubated in iron stained solution (1:1 potassium ferrocyanide:hydrochloric acid) for 3 minutes. Tissue was counterstained with nuclear fast red for 5 minutes. Iron is stained blue and nuclei are red/pink.

Imaging: The images were captured using a Zeiss Imager Z2 microscope with a 10x objective and an Axiocam 506 colour camera (Carl Zeiss, Oberkochen, Germany). Image analysis was carried out with the Zen 2 software (Carls Zeiss).

Immunocytochemistry

hPASMCs were seeded on 8 well chamber slides at 10,000 cells per well. Briefly, the cells were fixed using 100% freezing cold methanol for 10 minutes, followed by permeabilisation (0.2% Triton X-100 in PBS) for 5 minutes. The slides were then blocked with 1% BSA in PBS for an hour followed by incubation with mouse anti-α smooth muscle actin (SMA; R&D Systems, MAB 14106) rabbit anti-SM22 alpha (Abcam, ab14106) and mouse anti-MHC (smooth muscle myosin heavy chain; Abcam, ab603) another hour. A negative control without any primary antibody was also employed. Following four washes with 0.1% Tween-20 in PBS, the slides were incubated with 1:500 Alexa Flour 568 Goat anti-rabbit IgG (Invitrogen) and 1:500 Alexa Flour 488 Goat anti-mouse IgG (Invitrogen). The cells were further counterstained with DAPI and images captured using Leica SP5 inverted (Fast) confocal microscope at the FILM (Facility for Imaging by Light Microscopy) facility at Imperial College London.

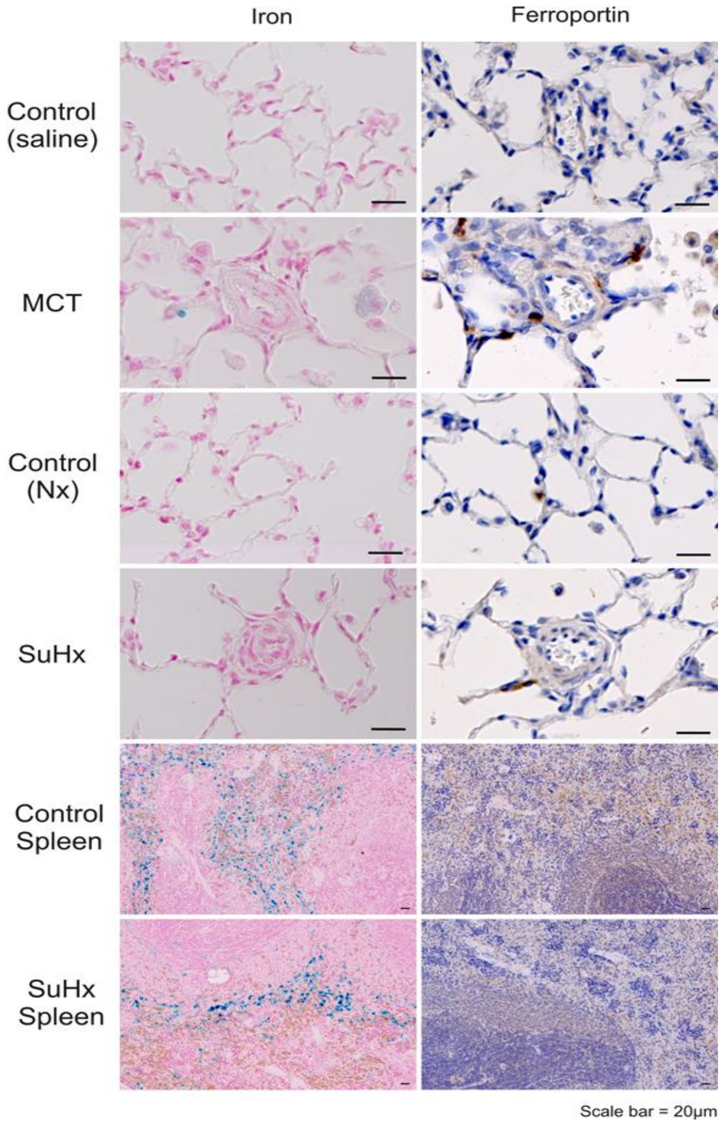

Supplementary Figure 1.

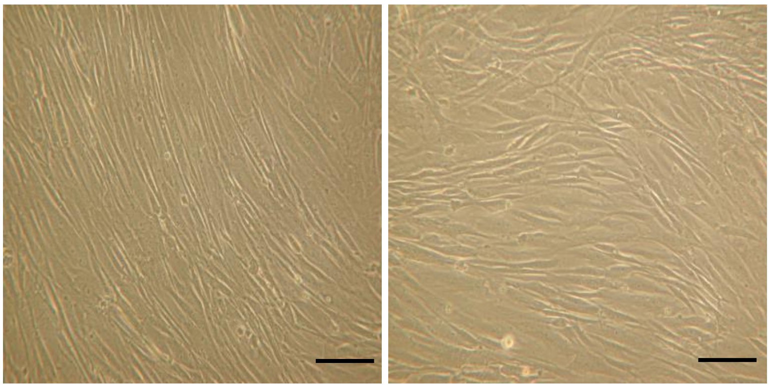

Supplementary Figure 2. *Phase contrast images of confluent hPASMCs, isolated from patients at the Royal Brompton Hospital. The cells were phased bright and showed a spindle-shaped appearance and formed bands of parallel cells. Scale bar = 10 µM.*

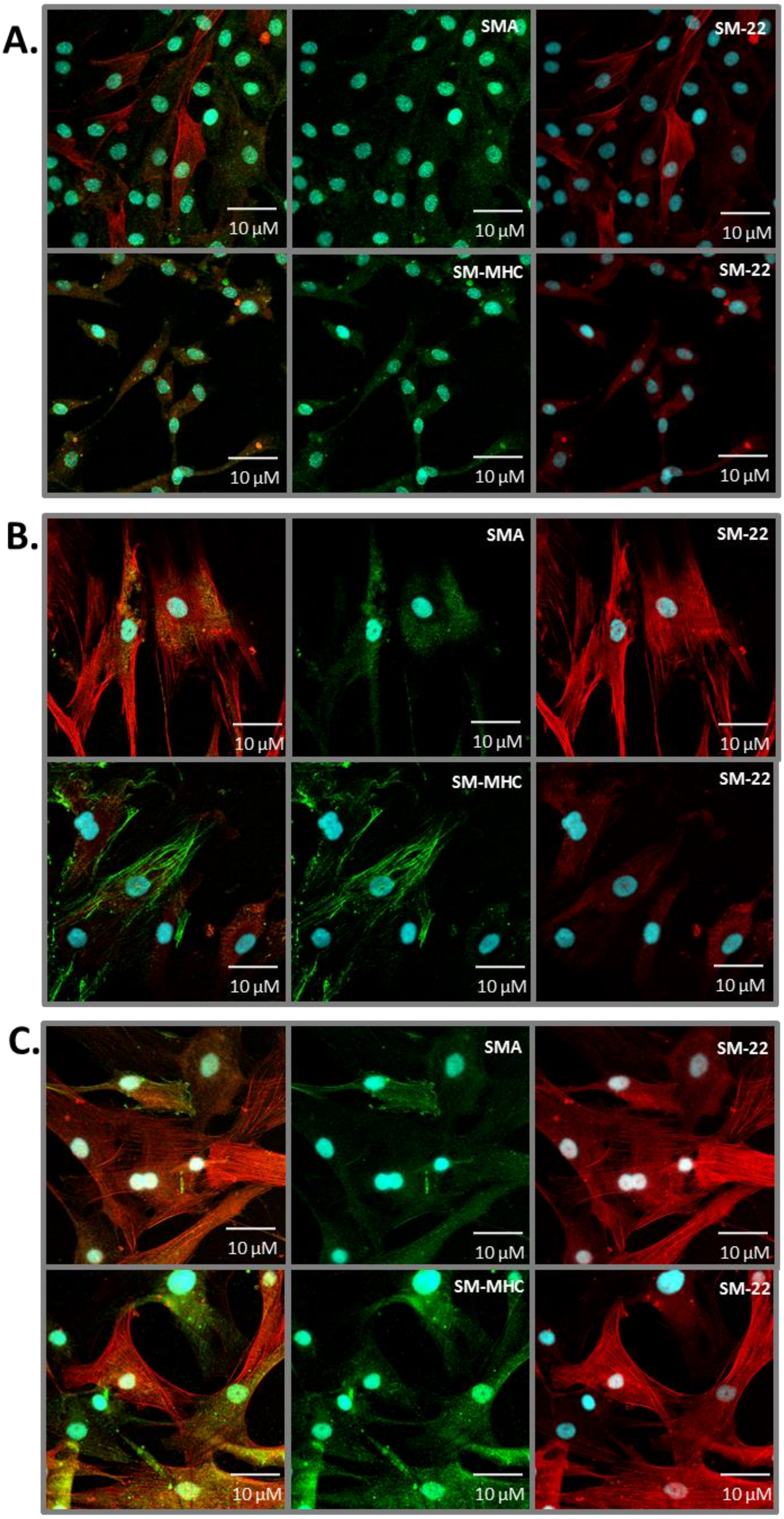

Supplementary Figure 3. *Confocal images of hPASMCs from three donors, (A) donor-5155, (B) donor-5174, and (C) donor-5164; grown in smooth muscle cell growth medium 2 (Promocell, Germany) or DMEM 15% FBS medium. Top 3 panels of each figure were immuno-stained with rabbit anti-SM22 alpha (red) and mouse anti- SMA (Smooth muscle actin). Bottom 3 panels were immuno-stained with rabbit anti-SM22 alpha (red) and anti-mouse MHC (smooth muscle myosin heavy chain; green). Scale bar = 10 µM*

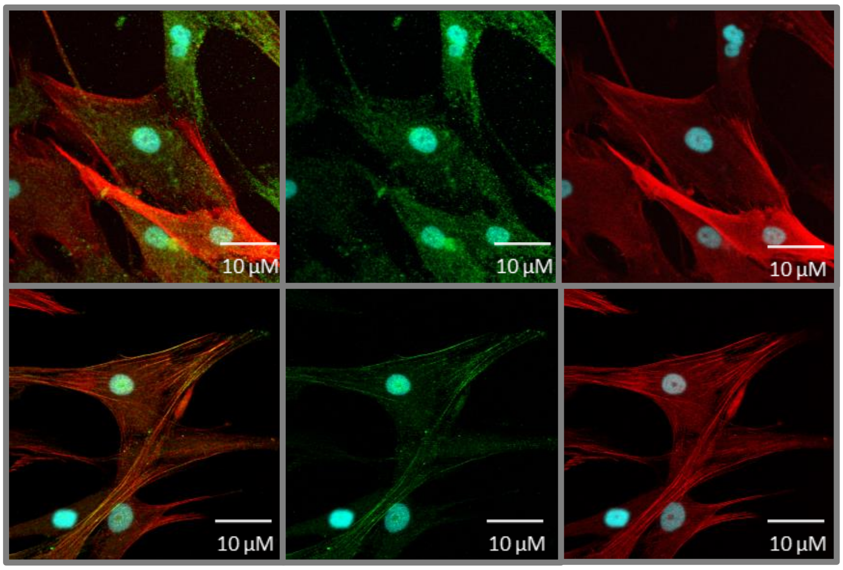

Supplementary Figure 4*. Confocal images of hPASMCs purchased from Promocell, Germany, grown in smooth muscle cell growth medium 2 (Promocell, Germany). Top 3 panels immuno-stained with rabbit anti-SM22 alpha (red) and mouse anti-SMA (Smooth muscle actin). Bottom 3 panels were immuno-stained with rabbit anti-SM22 alpha (red) and mouse anti-MHC (smooth muscle myosin heavy chain; green). Scale bar = 10 μM*
